# Network pharmacology analysis and clinical efficacy study of Fuzi Lizhong Decoction combined with XELOX plus sintilimab in the treatment of advanced gastric cancer

**DOI:** 10.1097/MD.0000000000044262

**Published:** 2025-09-12

**Authors:** Wei Zhang, Wei Hu, Jinxing Ji, Weipeng Liu, Lirong Deng, Yanfang Deng, Hushuang Yang, Xingying Zheng, Siying Le, Hongliang Zhang

**Affiliations:** a Department of Oncology, The Fourth Clinical Medical College of Xinjiang Medical University, Urumqi, Xinjiang, China; b Department of Oncology, College of Health Medicine, Three Gorges University, Yichang, Hubei, China; c Department of Oncology, Yichang Central People’s Hospital, Yichang, Hubei, China; d Department of Oncology, Three Gorges University Hospital of Traditional Chinese Medicine & Yichang Hospital of Traditional Chinese Medicine, Yichang, Hubei, China; e Department of Oncology, Hospital of Traditional Chinese Medicine Affiliated to Xinjiang Medical University, Urumqi, Xinjiang, China.

**Keywords:** clinical observation, Fuzi Lizhong Decoction, gastric cancer, network pharmacology, XELOX plus sintilimab

## Abstract

This study aimed to evaluate the clinical efficacy of Fuzi Lizhong Decoction (FZLZD) as an adjunct therapy for advanced gastric cancer (GC) when combined with the capecitabine plus oxaliplatin (XELOX) plus sintilimab regimen and to elucidate its potential therapeutic mechanisms through network pharmacology. Network pharmacology was employed to identify active ingredients of FZLZD and predict key targets and signaling pathways involved in GC treatment. GeneMANIA-based Functional Association analysis, core microRNA screening, and immunohistochemical analysis of core proteins were performed. Molecular docking was used to verify the direct regulatory effects of these traditional Chinese medicine monomers on the targets. FZLZD was found to have 732 targets associated with GC, involving processes such as protein phosphorylation, signal transduction, and gene transcription regulation. These targets were enriched in tumor- and metabolism-related pathways. The core proteins included protein tyrosine phosphatase non-receptor type 1, estrogen receptor 2, cytochrome P450 family 19 subfamily A member 1, and interleukin 6, while the core drug monomers included glypallichalcone, α-amyrin, deoxyharringtonine, and neokadsuranic acid B. Molecular docking results showed strong binding between the core targets and monomers. Clinical comparative studies have shown that compared with the XELOX plus sintilimab regimen alone, the combination of FZLZD and this regimen can significantly reduce traditional Chinese medicine symptom scores, lower tumor marker levels, and interleukin 6 levels in the treatment of advanced GC, and meaningfully improve health-related quality of life metrics. This study provides preliminary evidence that FZLZD, when combined with the XELOX plus sintilimab regimen, enhances the efficacy of the treatment for advanced GC. The results suggest that FZLZD may modulate key targets and signaling pathways, offering insights for further research and clinical application.

## 1. Introduction

Gastric cancer (GC) is a common malignant tumor of the digestive system, which can be caused by various factors such as *Helicobacter pylori* infection, dietary habits, family history, and genetic predisposition. Globally, the incidence and mortality of GC continue to rise.^[1,2]^ Early GC is often misdiagnosed as a digestive system disease due to its mild symptoms. Most cases are already in the advanced stage of GC when diagnosed and/or detected, missing the optimal treatment time.^[3,4]^ Currently, chemotherapy and immunotherapy are still the mainstream treatments for GC, among which the combination of capecitabine plus oxaliplatin (XELOX) chemotherapy and immunotherapy has been widely recognized. This combination therapy can effectively slow down the progression of the disease and is expected to prolong the patient’s life.^[5]^ However, combined therapy often leads to a series of drug toxicity and drug resistance in some GC patients.^[6,7]^

With the promotion and development of traditional Chinese medicine (TCM), its advantages have gradually gained clinical recognition and promotion worldwide. A large number of clinical studies have confirmed that the combination of TCM and Western medicine was more effective than a single drug in treating diseases. In the treatment of GC, the combination of TCM with chemotherapy has a significant synergistic effect, which can alleviate the discomfort caused by chemotherapy, improve the patient’s immunity and quality of life, and have broad application prospects.^[8,9]^ Fuzi Lizhong Decoction (FZLZD), a prescription, first appeared in the Song Dynasty about 1000 years ago. It was famous for warming the middle-jiao and tonifying the spleen and was used to treat spleen yang deficiency syndrome.^[10]^ FZLZD is composed of 5 key medicinal herbs, each with documented pharmacological effects. *Aconitum carmichaeli* (Fuzi) has anti-inflammatory and immune-enhancing properties. *Panax ginseng* (Renshen) strengthens the immune system and alleviates fatigue. *Atractylodes macrocephala* (Baizhu) improves digestion and immunity, while *Glycyrrhiza uralensis* (Gancao) reduces toxicity and supports liver function. *Zingiber officinale* (Ganjiang) has antioxidant and anti-inflammatory effects. Together, these components may alleviate chemotherapy side effects and enhance the effectiveness of chemotherapy and immunotherapy in treating GC. This study focused on 40 patients with advanced GC, to analyze the clinical efficacy of FZLZD combined with XELOX plus sintilimab therapy in the treatment of advanced GC.

We hypothesize that FZLZD, when used as an adjunct to the XELOX and sintilimab regimen, enhances the therapeutic effects of chemotherapy and immunotherapy in advanced GC patients by modulating key molecular targets and signaling pathways. We further hypothesize that this combination therapy can improve the patients’ clinical outcomes, including reducing tumor markers, improving quality of life, and enhancing immune function, while minimizing chemotherapy-induced side effects.

To further elucidate the mechanism of FZLZD enhancing XELOX plus sintilimab in the treatment of GC, this study explored its pharmacological mechanism through network pharmacology methods. Which was a relatively new research strategy for TCM formulas, and had been widely used for the elucidation of pharmacological mechanisms. By constructing a “Chinese medicine–monomer–target” network and using network topology methods to screen the core targets, the complex mechanism of action of TCM formulas can be systematically studied.^[11]^ This method provided a reference for revealing the pharmacological mechanisms of TCM syndromes and diseases.

## 2. Materials and methods

### 2.1. Acquisition of active ingredients and potential targets of FZLZD

This retrospective, exploratory study was approved by the Medical Ethics Committee of Yichang TCM Hospital on March 15, 2023, with approval number 2023004. FZLZD was composed of 5 TCMs including *A carmichaeli* Debx. (Fuzi), *P ginseng* C. A. Mey. (Renshen), *A macrocephala* Koidz. (Baizhu), *G uralensis* Fisch. (Gancao), and *Z officinale* Rosc. (Ganjiang). The chemical components of each Chinese medicine in FZLZD were obtained through the Traditional Chinese Medicine Systems Pharmacology Database (TCMSP) database (https://old.tcmsp-e.com/browse.php?qc=herbs), and based on oral bioavailability ≥ 30% and drug likeness ≥ 0.18, screen for Chinese herbal components with potential medicinal properties. Then, the typical SMILES numbers corresponding to the active ingredients were retrieved from the PubChem database (https://pubchem.ncbi.nlm.nih.gov/), and imported into Swiss Target Prediction (http://www.swisstargetprediction.ch/) to predict the target genes of TCM monomers. After removing duplicate target genes with a probability >0, the gene names were standardized on the uniport website (https://www.uniprot.org/) to obtain the active ingredient targets of the FZLZD drug monomer.

### 2.2. Target screening and protein interaction network construction of FZLZD in the treatment of GC

Using “gastric cancer” as the keyword, we searched for disease-associated genes in the databases OMIM (https://www.omim.org/), Genecards (https://www.genecards.org/), Drugbank (https://go.drugbank.com/), Disgenet (https://www.disgenet.com/), and TTD (https://db.idrblab.net/ttd/). The above disease-associated genes were merged and deduplicated, and the gene names were standardized on the uniport website. The FZLZD single-drug associated genes and disease target genes were imported into the Jvenn website (https://jvenn.toulouse.inrae.fr/app/example.html) to analyze the intersection genes (Drug Targets), and the drug targets were imported into STRING (https://cn.string-db.org/), the species was selected as “*Homo sapiens*” and with a confidence level of 0.7 for protein–protein interaction (PPI) network analysis.

### 2.3. Gene functional enrichment analysis of drug targets

Drug target genes were imported into DAVID (https://david.ncifcrf.gov/), and the database was used for gene ontology (GO) and Kyoto Encyclopedia of Genes and Genomes (KEGG) analysis, where GO analysis selected 3 analysis modules: molecular function, cellular components, and biological process. The results are visualized using the “ggplot2” package of R language (Vienna, Austria).

### 2.4. Construction of the network pharmacology map and core target screening of FZLZD in the treatment of GC

Correlation analysis was performed between drug targets and corresponding TCM monomers, and a “FZLZD–monomer–gene” network was constructed. The above network relationships were imported into Cytoscape 3.9.0 to construct a visualized TCM–monomer–gene network diagram. The MCC algorithm of the plug-in CytoHubba was used to screen the top 10 core monomers or core targets of TCM.

### 2.5. GMFA analysis and functional enrichment analysis of core targets

To further demonstrate the function of the screened core drug targets of FZLZD, GeneMANIA-based functional association (GMFA) analysis was performed on the core drug targets. Specifically, the 10 screened core targets were input into the STRING website, and the 10 related proteins with the strongest interactions were analyzed. All proteins were merged and subjected to GO and KEGG functional analysis again to explore the biological function of the core targets and their consistency with the targets function of FZLZD in treating GC.

### 2.6. Screening of microRNAs associated with core target genes

The core target genes were input into the miRDB website (https://mirdb.org/mirdb/index.html) to analyze the associated microRNAs, and the above microRNAs were intersected with the disease-associated microRNAs in the Genecards database. Then, the network of the above intersection microRNAs and the core genes was constructed, imported into Cytoscape 3.9.0 and a network diagram was constructed, and the top 10 core microRNAs were further screened through the Cytohubbo plug-in.

### 2.7. Analysis of core gene expression in gastric tissue

The expression levels of core targets in normal and GC tissue were analyzed on the Gene Expression Profiling Interactive Analysis website (http://gepia2.cancer-pku.cn/#index), and then the immunohistochemical data of different proteins were downloaded and analyzed on The Human Protein Atlas website (https://www.proteinatlas.org/) according to their expression differences.

### 2.8. Molecular docking

The screened core monomers of TCM were molecularly docked with the core proteins. The crystal structures of the core proteins were obtained from the PDB database (https://www.rcsb.org/), and the chemical structures of the core monomers (sdf format) were downloaded from the PubChem database (https://pubchem.ncbi.nlm.nih.gov/). Then, the core monomers of FZLZD were molecularly docked using Autodock4 and Autogrid4 software to calculate the affinity value of the best binding. The target proteins with binding ability to the compounds were screened, and the molecular docking results were displayed using Pymol software.

### 2.9. Diagnosis, staging, inclusion, and exclusion criteria of GC

The diagnostic criteria for GC were based on the “Guidelines for the Diagnosis and Treatment of Gastric Cancer (2023 Edition),”^[12]^ The staging of cancer followed the 8th edition staging guidelines published by the American Joint Committee on Oncology.^[13]^ According to the Guiding Principles for Clinical Research of New Chinese Medicines, the GC patients included in this study met the criteria for spleen and stomach deficiency and cold syndrome, as evidenced by symptoms such as epigastric pain, fatigue, poor appetite, pale tongue coating, and a thin or slippery pulse. The diagnosis of TCM syndrome types was performed by experienced clinicians specialized in TCM, following a standardized diagnostic approach. This approach involved a comprehensive assessment, including symptom identification (such as the presence of epigastric pain, fatigue, and poor appetite) and tongue and pulse examination: 2 key diagnostic elements in TCM. Although there is an element of subjective judgment involved, the diagnosis was made based on well-established diagnostic criteria and clinical experience, ensuring consistency and reliability. Additionally, multiple TCM experts reviewed and confirmed the syndrome types to minimize individual bias and ensure accurate classification. The inclusion and exclusion criteria were based on the references.^[12]^

### 2.10. Clinical study plan of FZLZD combined with XELOX plus sintilimab regimen for the treatment of GC

This retrospective, exploratory study was approved by the Medical Ethics Committee of Yichang TCM Hospital on March 15, 2023, with approval number 2023004. From October 2023 to September 2024, 40 GC patients who met the inclusion criteria and received treatment at Yichang TCM Hospital were included in the study. The sample size was calculated based on a power analysis, aiming to achieve a 95% confidence level and 80% power, ensuring that the study had enough statistical strength to detect significant differences. The primary outcome for sample size estimation was the difference in Karnofsky Performance Status (KPS) scores between the 2 groups. The 40 patients were randomly divided into 2 groups based on the principle of computer-generated random allocation. Specifically, patients were assigned to either the control group or the observation group using a computer program that generated random numbers (simple randomization method). This process was done by an independent statistician who had no involvement in patient treatment or outcome evaluation. The control group was treated with XELOX chemotherapy combined with sintilimab, and the specific treatment plan was referred to the literature.^[9]^ The observation group was treated with FZLZD in addition to the control group treatment plan. The formula of FZLZD includes: Renshen (10 g), Baizhu (10 g), Ganjiang (10 g), Fuzi (10 g), and Gancao (5 g). Boil according to standard procedures, with a fixed dose of 200 mL per serving, and store at a low temperature after extracting air. The patient took the medication 3 times a day, with 200 mL each time, for 21 days.

### 2.11. Clinical efficacy testing

The clinical efficacy was evaluated after 2 cycles (42 days) of treatment. The evaluation criteria for therapeutic effects are based on the “Guiding Principles for Clinical Research of New Chinese Medicines.” Significant improvement: after treatment, the score of TCM symptoms decreased by ≥ 70%. Partial improvement: after treatment, the TCM symptom score decreased by 30% to 70%. No improvement: there was no change in the TCM symptom score after treatment. Total improvement rate/% = (significant improvement cases + partial improvement cases)/total cases × 100%.

### 2.12. Comparison of TCM syndrome scores and KPS scores

Before and after treatment, the TCM symptom scores of the 2 groups of patients were compared and analyzed according to the “Guiding Principles for Clinical Research of New Chinese Medicines” symptom grading scale. The higher the score, the more severe the symptoms. At the same time, KPS was used to evaluate the health status of the patients before and after treatment. A score of <50 was considered dependent, meaning that the patient needed help from others; 50 to 80 was considered semi-dependent, meaning that the patient could take care of himself; and 80 to 100 was considered non-dependent, meaning that the patient could take care of himself.

### 2.13. Detection of tumor markers and interleukin 6 (IL6) expression levels

One month before the start of treatment and after 2 cycles of treatment, patients were required to draw blood samples in a fasting state. These samples were tested by enzyme-linked immunosorbent assay to determine the expression levels of carcinoembryonic antigen, carbohydrate antigen 242, carbohydrate antigen 724, and IL6 in the serum.

### 2.14. Statistical methods

The data were analyzed using SPSS 22.0 statistical software (IBM Corporation, Armonk). For continuous variables with a normal distribution, independent *t* tests were used to compare the 2 groups, while Mann–Whitney *U* tests were applied to non-normally distributed data. Chi-square tests were used for categorical data. For pre- and post-treatment comparisons within the same group, a paired *t* test was used, and if the data was not normally distributed, the Wilcoxon signed-rank test was employed. A *P*-value of <.05 was considered statistically significant. Effect sizes (Cohen *d*) were calculated where applicable, and Bonferroni corrections were applied for multiple comparisons to control for type I error.

## 3. Results

### 3.1. Active compounds identified in FZLZD and their putative targets

In the TCMSP database, a total of 142 Chinese medicine monomer components were screened out according to drug-like properties and bioavailability, including 19 from Fuzi, 10 from Ganjiang, 21 from Renshen, 7 from Baizhu, and 85 from Gancao. The above monomers corresponded to a total of 1025 target genes.

### 3.2. Analysis of genes associated with the XELOX plus sintilimab regimen for the treatment of advanced GC and construction of the Venn diagram

**Figure 1.**
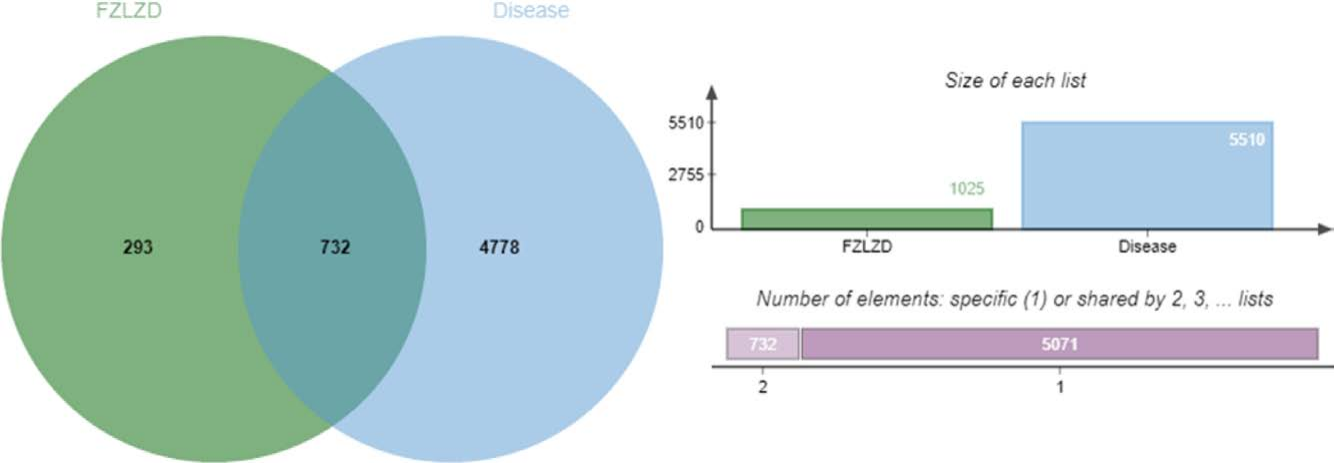
Venn diagram of FZLZD and disease-related targets. FZLZD = Fuzi Lizhong Decoction.

In 5 disease databases, 5519 disease-associated genes were obtained by using the keywords “gastric cancer” or “chemotherapy-induced gastrointestinal injury,” including 77 lncRNA genes, 241 microRNA genes, and 5201 protein-coding genes. By constructing a Venn diagram between drugs and disease genes, it was found that there were a total of 732 intersecting genes (Fig. 1).

### 3.3. Analysis of PPI between associated targets

The intersection targets were input into the STRING website to analyze the PPI relationship and construct a visual PPI network. The PPI results showed that proteins such as AKT1, TNF, and IL6 had the highest degree of interaction (Table 1).

**Table 1:** Top 10 therapeutic targets information with degree values.

Name	Degree	Betweenness centrality	Closeness centrality
AKT1	370	0.052509178	0.667582418
TNF	327	0.03580267	0.642857143
IL6	324	0.039079698	0.642290749
SRC	303	0.056001983	0.625214408
STAT3	281	0.020093835	0.615189873
EGFR	281	0.025178993	0.616751269
ALB	275	0.031705273	0.613636364

### 3.4. GO enrichment and KEGG pathway analysis

**Figure 2.**
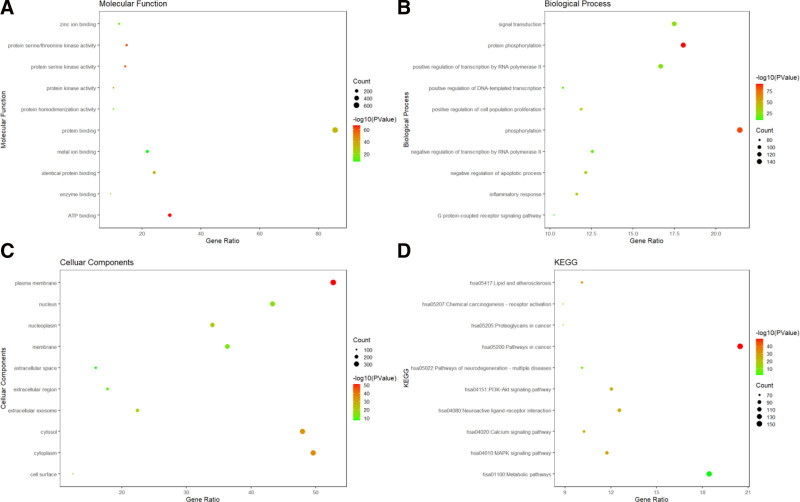
GO and KEGG analysis of intersection targets (A), molecular functional analysis (B), biological processes analysis (C), cellular components analysis (D), and KEGG analysis. GO = Gene Ontology, KEGG = Kyoto Encyclopedia of Genes and Genomes.

The GO analysis results of intersecting targets indicate that their biological processes were mainly related to phosphorylation, protein phosphorylation, signal transduction, and positive regulation of RNA polymerase II transcription. Molecular functions were closely related to protein binding and ATP binding. Cellular components analysis shows that they were closely related to membrane proteins, cytoplasm, cytosol, nucleus, etc. The KEGG analysis results of the intersection targets indicated that their gene functions are mainly related to tumors and metabolic pathways (Fig. 2).

### 3.5. Construction of network pharmacology diagram and screening of core targets for TCM–monomer–gene compound

**Figure 3.**
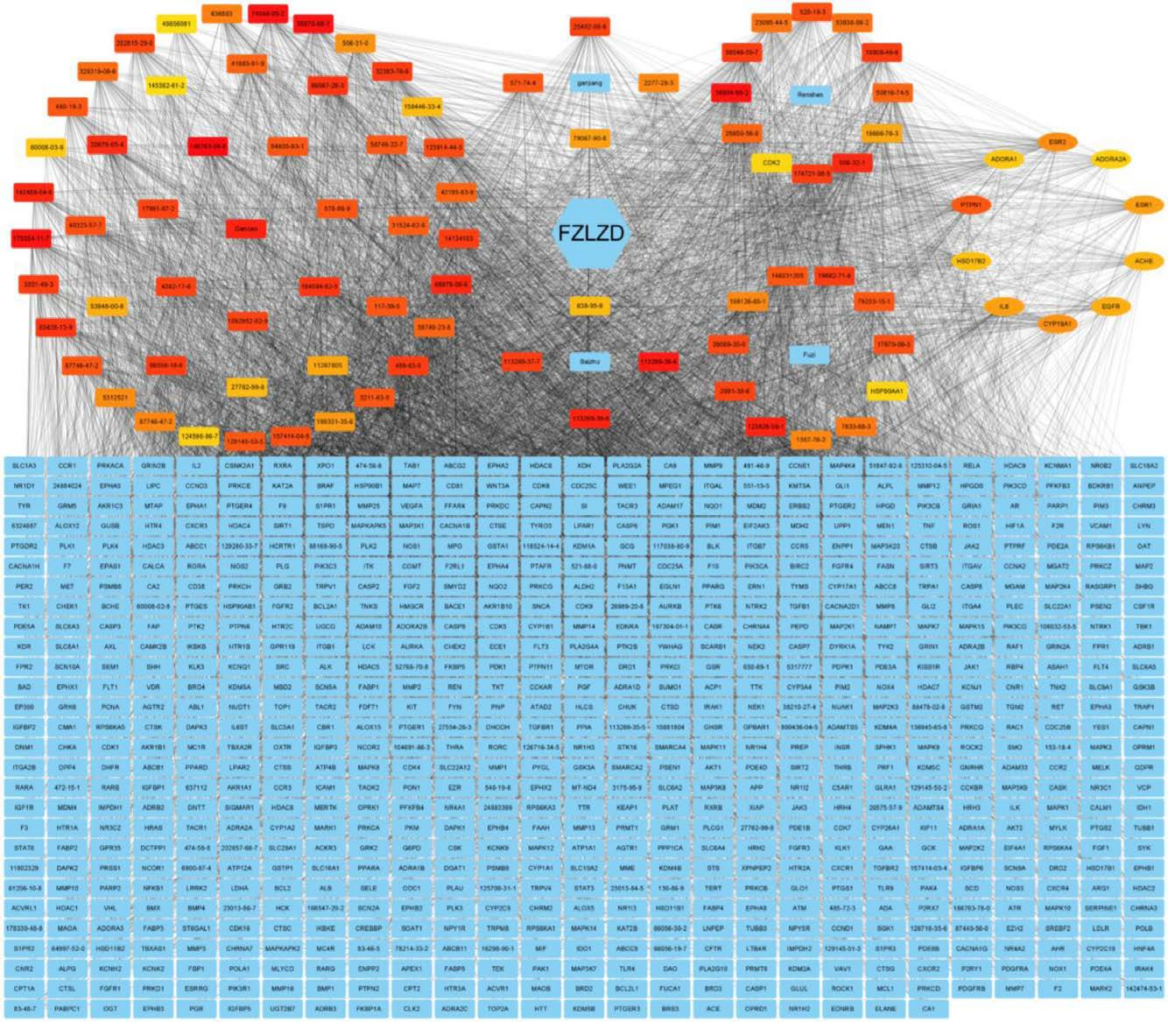
Network diagram of FZLZD compound. FZLZD = Fuzi Lizhong Decoction.

The construction of the compound network pharmacology diagram of FZLZD is shown in Figure 3. The network diagram has a total of 846 nodes and 7292 edges, with an average proximity of 17.239.

The MCC algorithm of the CytoHubba plugin in Cytoscape 3.9.0 was used to further screen the core monomers and core target genes of TCM. The core monomers are shown in Table 2, and the core target genes are shown in Table 3.

**Table 2:** Top 10 in network ranked by MCC method.

Rank	CAS number	Name	Source	Score
1	146763-58-8	Glypallichalcone	Gancao	94
2	175554-11-7	3’-Hydroxy-4’-O-Methylglabridin	Gancao	89
2	59870-68-7	Glabridin	Gancao	89
2	74046-05-2	3’-Methoxyglabridin	Gancao	89
2	36804-95-2	Deoxyharringtonine	Renshen	89
6	638-95-9	α-Amyrin	Baizhu	88
7	113269-35-5	8β-ethoxy atractylenolide Ⅲ	Baizhu	87
7	123828-59-1	Neokadsuranic acid B	Fuzi	87
10	142488-54-8	glyasperin B	Gancao	86
10	68978-09-6	2-[(3R)-8,8-dimethyl-3,4-dihydro-2H-pyrano[6,5-f]chromen-3-yl]-5-methoxyphenol	Gancao	86

**Table 3:** Top 10 in network ranked by MCC method.

Rank	CAS number	Score	Rank	CAS number	Score
1	PTPN1	78	6	EGFR	56
2	ESR2	68	7	ACHE	55
3	CYP19A1	66	8	HSD17B2	52
4	IL6	64	9	ADORA1	51
5	ESR1	64	10	ADORA2A	48

CYP19A1 = Cytochrome P450 family 19 subfamily A member 1, ESR2 = estrogen receptor 2, IL6 = interleukin 6, PTPN1 = protein tyrosine phosphatase non-receptor type 1.

### 3.6. GMFA analysis and functional prediction of core genes

**Figure 4.**
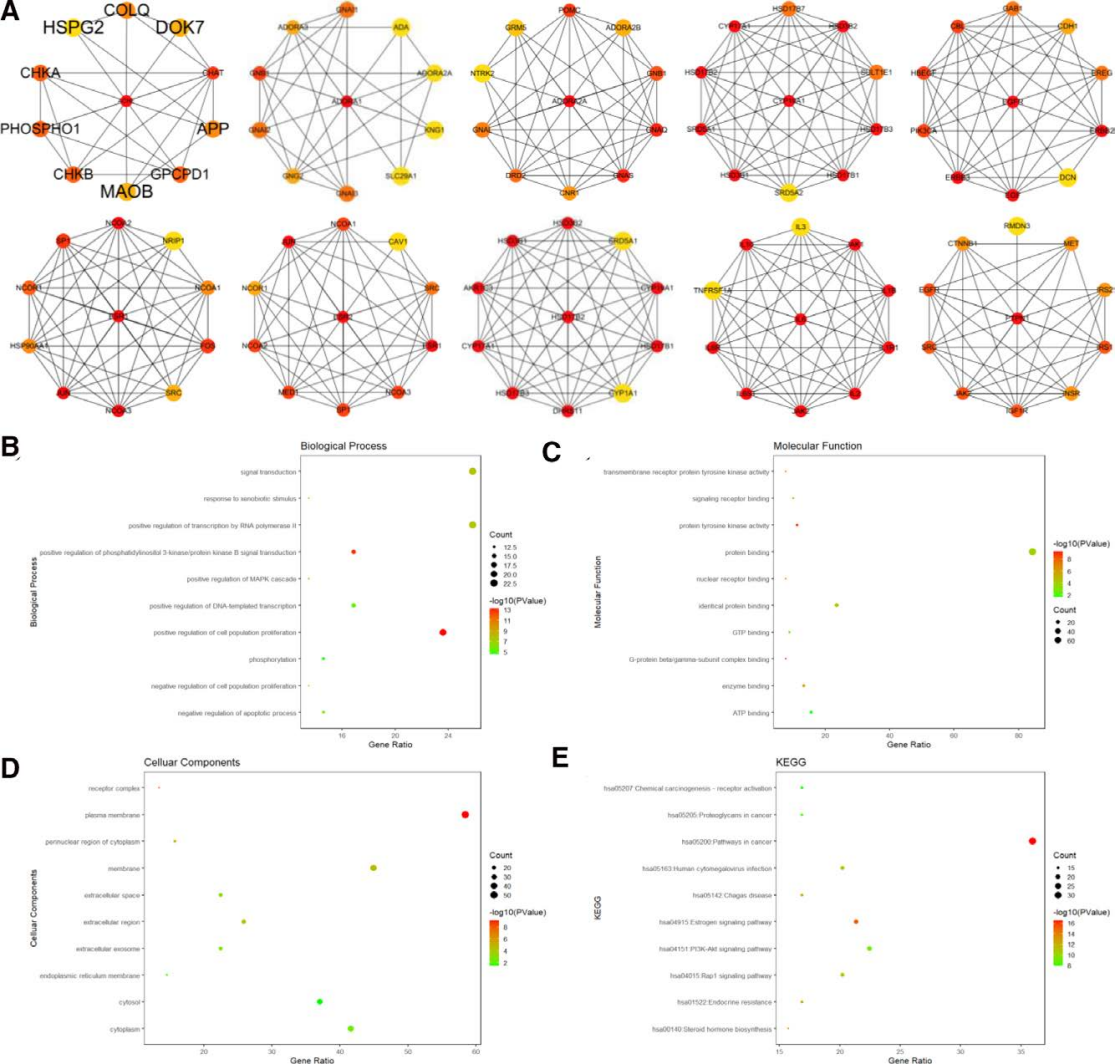
GMFA analysis and function re-analysis. (A) GMFA analysis; (B) biological processes analysis of GMFA-ED; (C) molecular functional analysis of GMFA-ED; (D) cellular components analysis of GMFA-ED; (E) KEGG analysis of GMFA-ED. GMFA = GeneMANIA-based Functional Association, GMFA-ED = GMFA extended database, KEGG = Kyoto Encyclopedia of Genes and Genomes.

Based on the functional association network analysis of the top 10 hub targets of GMFA, an extended potential therapeutic target database (GMFA-ED) for FZLZD against GC was created. This integration generated a total of 110 genes in the extended database, providing a more comprehensive perspective for identifying potential therapeutic targets for FZLZD combined with the XELOX plus sintilimab regimen for the treatment of advanced GC (Fig. 4A). The above 110 genes were analyzed again using the DAVID database for GO and KEGG analysis. The biological processes of the enriched genes were mainly related to signal transduction, positive regulation of cell population proliferation, and positive regulation of RNA polymerase II transcription. The molecular functions of the enriched genes were mainly closely related to protein binding. Cell components analysis showed that they were closely related to plasma membrane, membrane, cytoplasm, and cytosol. KEGG analysis showed that the functions of the enriched genes were mainly closely related to tumor pathways (Fig. 4B–E).

### 3.7. Screening of microRNAs targeting core genes

**Figure 5.**
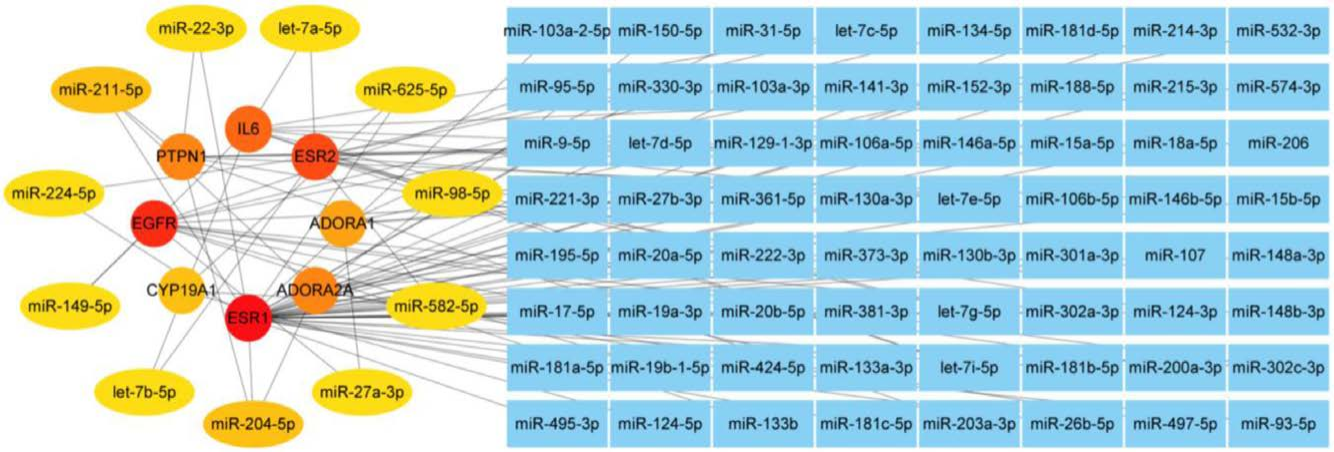
Construction of microRNA network and screening of core microRNAs.

As mentioned above, 241 microRNAs were involved in the regulation process of XELOX plus sintilimab in the treatment of advanced GC. To analyze which microRNAs were most related to the FZLZD combined with the XELOX plus sintilimab regimen in the treatment of advanced GC, we performed an association analysis between the 10 core target genes and the above 241 microRNAs and constructed a network diagram. The results of the MCC algorithm based on the Cytohubbo plug-in showed that microRNAs such as miR-22-3p, miR-211-5p, miR-224-5p, miR-149-5p, miR-204-5p, miR-27-3p, miR-582-5p, miR-98-5p, miR-625-5p, let-7a-5p, and let-7b-5p, as cores, respectively/synergistically regulated the biological process of FZLZD combined with the XELOX plus sintilimab regimen for the treatment of advanced GC (Fig. 5).

### 3.8. Analysis of gastric tissue expression of core genes

**Figure 6.**
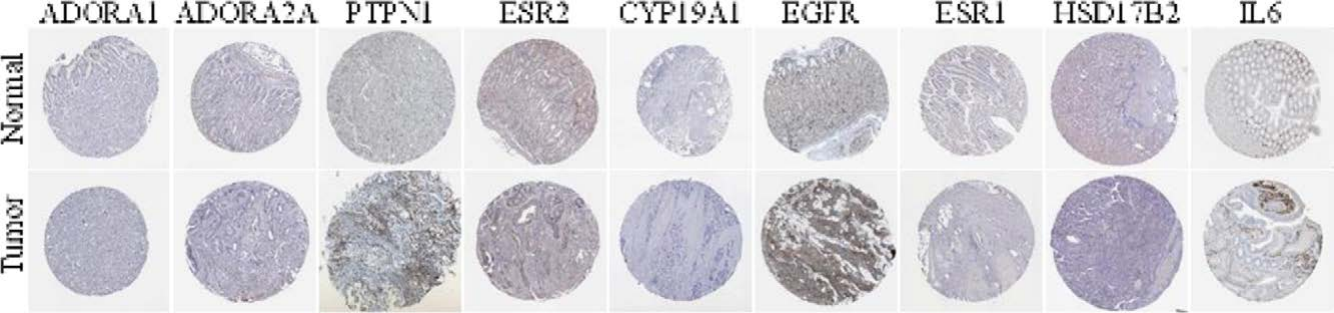
Immunohistochemical analysis of core targets in normal and gastric cancer tissues.

Analysis of the expression of 10 core targets in normal and GC tissues by the Gene Expression Profiling Interactive Analysis website showed that the above targets were upregulated to varying degrees in GC. Based on this, the immunohistochemical differences of gastric tissue with different targets were analyzed on The Human Protein Atlas website (Fig. 6).

### 3.9. Molecular docking

**Figure 7.**
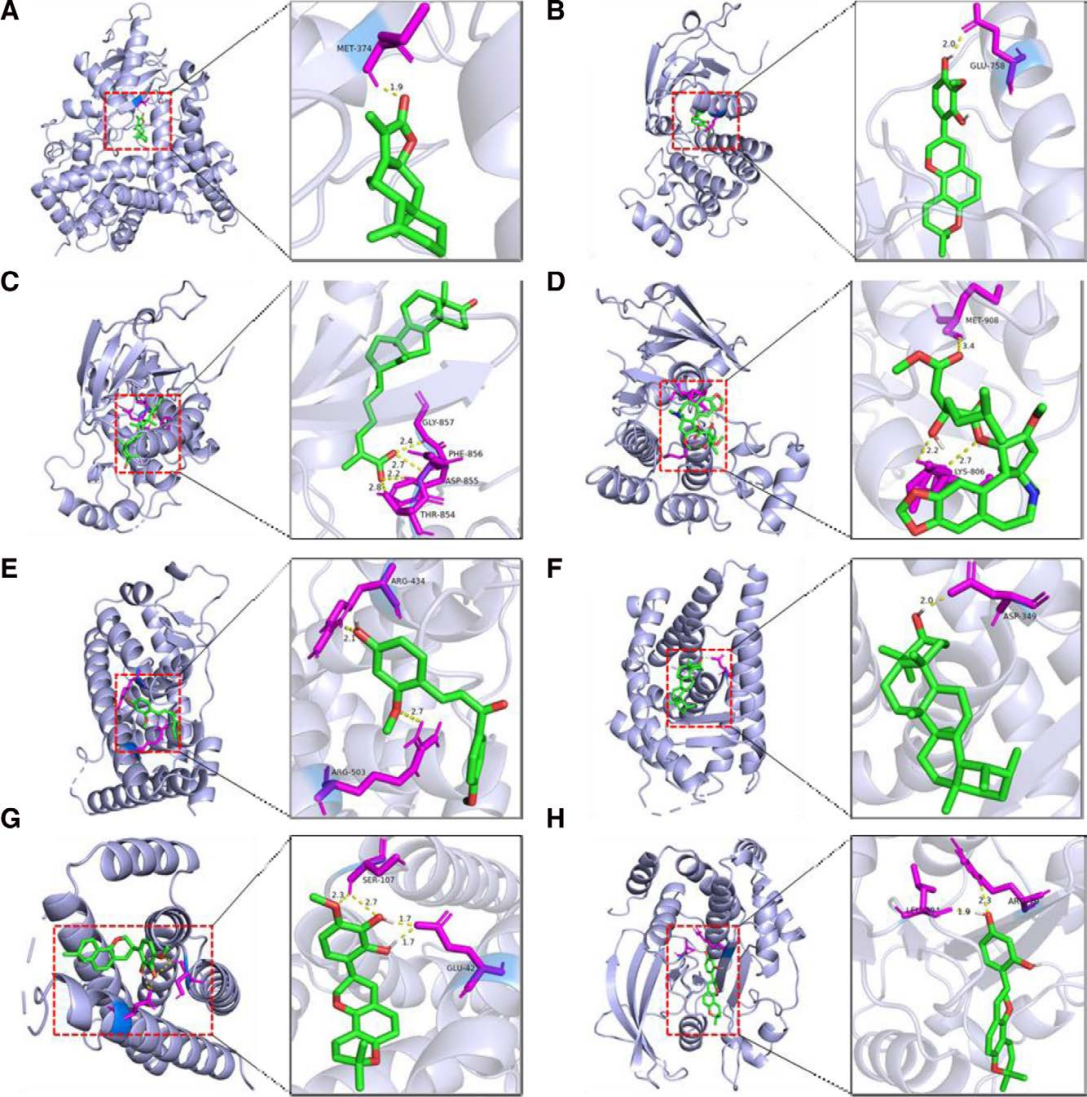
Molecular docking of typical monomers and core targets. (A) CYP19A1 versus 8β-ethoxy atractylenolide Ⅲ; (B) EGFR versus 3“-methoxyglabridin; (C) EGFR versus neokadsuranic acid B; (D) EGFR versus deoxyharringtonine; (E) ESR1 versus glypallichalcone; (F) ESR2 versus α-amyrin; (G) IL6 versus 3”-hydroxy-4‘-O-methylglabridin; (H) PTPN1 versus glabridin. CYP19A1 = cytochrome P450 family 19 subfamily A member 1, ESR2 = estrogen receptor 2, PTPN1 = protein tyrosine phosphatase non-receptor type 1

Based on the results of compound network pharmacology, the top 6 core targets were selected for molecular docking with the top 8 core monomers of TCM. The PDB numbers of the crystal structures of the core targets were cytochrome P450 family 19 subfamily A member 1 (5jkv), EGFR (8a27), ESR1 (6vpf), estrogen receptor 2 (ESR2) (3oll), IL6 (1alu), and protein tyrosine phosphatase non-receptor type 1 (PTPN1) (8skl), respectively. The highest free energy results of molecular docking are shown in Table 4. The average binding energy of 48 dockings was as high as −5.99 kJ/mol, indicating that the interaction between the core target and the core drug monomer was strong. The typical molecular docking results of each target or each monomer are shown in Figure 7.

**Table 4:** Molecular docking results (kJ/mol).

	CYP19A1	EGFR	ESR1	ESR2	IL6	PTPN1
3’-Hydroxy-4’-O-Methylglabridin	-6.23	-6.97	-6.38	-4.96	-7.03	-6.57
3’-Methoxyglabridin	-6.07	-7.19	-5.83	-6.07	-5.51	-5.47
α-Amyrin	-6.05	-6.73	-6.54	-7.71	-6.47	-6.42
8β-ethoxy atractylenolide Ⅲ	-7.27	-6.44	-7.49	-6.81	-5.00	-7.20
Neokadsuranic acid B	-6.13	-9.05	-5.49	-6.66	-5.91	-6.01
Deoxyharringtonine	-3.61	-4.57	-2.86	-3.25	-3.30	-4.43
Glabridin	-6.40	-7.67	-6.21	-6.13	-6.21	-6.20
Glypallichalcone	-5.24	-6.17	-5.91	-5.52	-4.25	-5.75

CYP19A1 = cytochrome P450 family 19 subfamily A member 1, ESR2 = estrogen receptor 2, IL6 = interleukin 6, PTPN1 = protein tyrosine phosphatase non-receptor type 1.

### 3.10. Comparison of clinical efficacy of FZLZD combined with XELOX plus sintilimab regimen in the treatment of advanced GC

The clinical efficacy of FZLZFD combined with the XELOX plus sintilimab regimen in the treatment of advanced GC showed that the overall improvement rate of the observation group after treatment was as high as 95%, which was significantly higher than that of the control group (*P* < .05) (Table 5).

**Table 5:** Comparison of therapeutic efficacy between observation and control groups.

Group	Number	Significant improvement	Partial improvement	No improvement	Overall improvement
Control group	20	0	12 (0.7)	8 (0.3)	60%
Observation group	20	2 (0.1)	17 (0.85)	1 (0.05)	95%
*P*-value					.026

### 3.11. Comparison of TCM syndrome points and KPS scores

The results of TCM syndrome points showed that after treatment with the XELOX plus sintilimab regimen, the TCM syndrome points of the patients were lower than that before treatment, and the reduction in the observation group after combined treatment with FZLZD was greater than that in the control group (*P* < .05). At the same time, the comparison of KPS scores showed that the KPS scores of the treatment group and the observation group increased significantly after treatment, and the differences were statistically significant compared with the same group before treatment (*P* < .01), and the improvement effect of the treatment group was statistically significant compared with the control group (*P* < .01) (Table 6).

**Table 6:** Comparison of TCM syndrome points and KPS scores between the 2 groups of gastric cancer patients before and after treatment ($\bar{x}\pm s$).

Group	Number	Time	TCM syndrome points	KPS scores
Control group	20	Before treatment	20.2 ± 2.35	66.45 ± 2.73
		After treatment	12.1 ± 3.04*	73.15 ± 2.37*
Observation group	20	Before treatment	20.1 ± 2.05	64.75.1 ± 2.45
		After treatment	10.05 ± 3.59*†	77.1 ± 3.24†‡

KPS = Karnofsky Performance Status, TCM = traditional Chinese medicine.

* Compared with the control group before treatment, *P* < .05.

† Compared with the control group after treatment, *P* < .05.

‡ Compared with the observation group before treatment, *P* < .05.

### 3.12. Analysis of tumor markers and IL6 expression levels

Tumor marker analysis showed that after treatment with the XELOX plus sintilimab regimen, the levels of tumor markers carcinoembryonic antigen, carbohydrate antigen 242, carbohydrate antigen 724, and inflammatory cytokine IL6 in the patient’s serum decreased compared to before treatment, and the observation group combined with FZLZD showed a greater decrease than the control group (*P* < .05) (Table 7).

**Table 7:** Comparison of tumor markers and IL6 levels in 2 groups of patients with advanced gastric cancer before and after treatment.

Group	Number	Time	CEA (ng/mL)	CA242 (U/mL)	CA724 (U/mL)	IL6 (pg/mL)
Control group	20	Before treatment	72.00 ± 2.68	29.27 ± 7.34	20.68 ± 9.36	27.21 ± 5.03
		After treatment	46.57 ± 6.29*	17.97 ± 2.21*	12.33 ± 4.48*	21.89 ± 1.98*
Observation group	20	Before treatment	72.26 ± 3.54	24.33 ± 6.24	19.31 ± 12.15	29.29 ± 4.78
		After treatment	19.67 ± 6.91†‡	12.79 ± 3.07†‡	7.38 ± 1.47†‡	20.35 ± 1.14*†

CEA = carcinoembryonic antigen, CA242 = carbohydrate antigen 242, CA724 = carbohydrate antigen 724, IL6 = interleukin 6.

* Compared with the control group before treatment, *P* < .05.

† Compared with the control group after treatment, *P* < .05.

‡ Compared with the observation group before treatment, *P* < .05.

## 4. Discussion

GC is a common malignant tumor, which is characterized by a high incidence rate, high mortality, and a younger incidence group in China.^[14]^ The pathogenesis of GC is relatively complex, and multiple factors such as *H pylori* infection, genetic factors, dietary habits, and autoimmunity may all contribute to its occurrence. Currently, surgical treatment, radiotherapy, chemotherapy, molecular targeted therapy, and other methods are comprehensively used in the treatment of GC. For patients with advanced GC, although chemotherapy and immunotherapy can partially prolong their survival time, there are still disadvantages such as drug tolerance and susceptibility to adverse reactions, which seriously affect drug efficacy and patient quality of life.^[15]^ In comparison, TCM’s ability to prevent and treat complex diseases has been increasingly confirmed and applied in clinical practice. For GC, many TCMs can improve patients’ immunity, alleviate adverse reactions to chemotherapy, and improve the quality of life. Their combination with chemotherapy drugs can play a good role in reducing toxicity and enhancing efficacy.^[16]^

In the field of TCM, GC can be classified as symptoms such as “dysphagia,” “nausea,” “abdominal mass,” and “abdominal lump.”^[17]^ FZLZD was first published in Treatise on 3 categories of pathogenic factors. Its formula contains 5 Chinese medicinal herbs: Fuzi, Renshen, Ganjiang, Baizhu, and Gancao. Among them, Fuzi can dispel cold, relieve pain, and has a warming effect on the yang qi of the heart, spleen, stomach, and kidneys; Baizhu has the effect of tonifying Qi and invigorating spleen, promoting urination and eliminating dampness; Ganjiang helps to warm middle-jiao to dispel cold; Rensheng can reinforce vital energy. Gancao (prepared) can tonifying the spleen and tonifying qi, relieve acute pain, and coordinate the effects of other medicinal herbs. Various drugs work synergistically to achieve the effects of replenishing weak yang energy, warming internal organs, and dispelling cold. Previous studies have shown that FZLZD can significantly enhance the immune response of patients in the treatment of advanced GC, effectively reduce the toxic side effects caused by radiotherapy and chemotherapy, relieve stomach pain, and significantly improve the quality of life.^[10,18,19]^ However, currently, there were no reports on the combination of FZLZD and XELOX plus sintilimab regimen for the treatment of GC, our study showed that compared with the XELOX plus sintilimab regimen alone, the combination of FZLZD and XELOX plus sintilimab regimen can significantly reduce TCM syndrome scores, serum tumor marker levels, and IL6 expression levels in the treatment of GC, and increase KPS scores, indicating that the combined use of FZLZD can significantly improve the symptoms of patients with advanced GC and improve their quality of life. This further confirmed the important role of TCM combined with modern medicine in the treatment of complex diseases.

This study used network pharmacology methods to retrieve and screen 142 active ingredients and 732 targets of FZLZD from the TCMSP database. The above targets mainly affect RNA transcription regulation and protein phosphorylation processes through signal transduction pathways, and then treat GC by inhibiting the tumor pathways. The construction of a compound network pharmacological map based on “FZLZD–herb–monomer–gene” and the screening of core monomers and targets showed that the drug monomers that exert core effects include glypallichalcone (derived from Gancao), deoxyharringtonine (derived from Renshen), α-amyrin (derived from Baizhu), and neokadsuranic acid B (derived from Fuzi), etc. The corresponding core targets include PTPN1, ESR2, cytochrome P450 family 19 subfamily A member 1, IL6, ESR1, and EGFR, etc. Molecular docking results also showed that the above monomers have good binding affinity to the targets. It was worth noting that the single drug component of Ganjiang does not appear in the top 10 core monomers of TCM. This drug may only play an auxiliary role in this study. In addition, there are 2 significant problems with searching for TCM monomers based on the TCMSP database. First, there were as many as 85 Gancao components that meet bioavailability and drug-like properties, indicating that this method ignored drug dosage and proportion, resulting in apparently effective drugs. Too many ingredients reduced the credibility of the results; secondly, it was well known that the main active ingredient of Renshen is ginsenosides,^[20]^ but it was excluded from the effective components of this study due to its low oral bioavailability. In addition, it showed that there was room for improvement in traditional network pharmacology drug screening methods and standards. Integrated analysis of multiple databases or direct analysis of blood components of drugs may be reasonable ways to solve these problems.

In this study, 6 core targets were screened by compound network pharmacology, and molecular docking was performed with 8 core monomers. The docking free energies were low, indicating that the drug had a strong binding effect on the above targets. Among them, PTPN1 and EGFR were important members of the protein tyrosine phosphatase family and play crucial roles in regulating signaling molecules involved in various cellular processes, including cell growth, differentiation, mitosis cycle, and carcinogenesis.^[21,22]^ As estrogen receptor transcription factors, ESR1 and ESR2 can also form homo/heterodimers in the nucleus. The 2 proteins played an important role in growth, metabolism, and tumorigenesis by regulating the transcription of estrogen-induced genes. And were closely related to breast cancer, cancer, osteoporosis, and other diseases.^[23]^ In addition, estrogen receptors and NF-κB inhibit each other in a cell type-specific manner, thereby reducing NF-κB activity and inhibiting its mediated IL6 transcription.^[24]^ IL6 is an oncogenic inflammatory factor that plays an important role in chronic tumor inflammation and maintaining the tumor microenvironment. Overexpression of IL6 was closely related to poor tumor prognosis.^[25]^ This study showed that after treatment in the observation group, the level of IL6 in the serum of patients decreased, suggesting that the combined effect of FZLZD with XELOX plus sintilimab regimen in the fight against GC may be related to the reduction of the expression of IL6.

GMFA analysis was used to enrich the discoveries of network pharmacology by exploring the functional associations of hub targets.^[26]^ This method involved discovering 10 additional genes for each hub gene and prioritizing genes with the strongest associations in the gene–gene network.^[27]^ A GMFA-ED was constructed based on the core and their associated genes. GO and KEGG analysis of GMFA-ED showed that the gene functions screened by GMFA were consistent with the drug targets, further demonstrating the important synergistic effect of core genes in the treatment of advanced GC with the combination of FZLZD combined with XELOX plus sintilimab regimen.

MicroRNAs play an important regulatory role in the occurrence and development of tumors.^[28]^ This study screened out 11 important microRNAs targeting core genes through association analysis between microRNAs and core genes. Existing research has shown that these 11 microRNAs are closely related to the occurrence and development of GC.^[29–31]^ Genes such as miR-211-5p and miR-224-5p may also be involved in the process of FZLZD synergistically enhancing the XELOX plus sintilimab regimens in the treatment of GC, but the specific mechanism needed to be further confirmed and clarified.

### 4.1. Study limitations

This study has several limitations that should be considered when interpreting the results. First, the sample size of 40 patients is relatively small, which may limit the generalizability of the findings. A larger cohort would provide more robust data and increase the reliability of the conclusions drawn. Second, this study did not include a long-term follow-up period. As a result, the long-term effects of FZLZD combined with XELOX and sintilimab on survival rates and disease recurrence remain unclear. Further research with extended follow-up would be necessary to evaluate the sustained efficacy and potential long-term benefits of this combination therapy. Additionally, due to the retrospective nature of this study, there may be biases in patient selection, and prospective randomized controlled trials are needed to confirm the findings and provide stronger evidence of the treatment’s clinical efficacy.

## 5. Conclusion

In summary, the observations of this study showed that the combination of FZLZD and XELOX plus sintilimab in the treatment of advanced GC can significantly reduce TCM syndrome scores, reduce serum tumor marker levels and IL6 expression, and enhance the therapeutic effect of advanced GC. Network pharmacology research showed that FZLZD mainly reduces the expression of proteins such as PTPN1, EGFR, and IL6 by affecting cell signal transduction pathways and protein phosphorylation modifications, thereby delaying tumor cell proliferation, promoting tumor cell death, synergistically enhancing the efficacy of XELOX plus sintilimab regimen in the treatment of advanced GC, and ultimately improving patient quality of life. This study provided a new clinical medication reference for the treatment of advanced GC.

## Author contributions

**Conceptualization:** Wei Zhang, Wei Hu, Hongliang Zhang .

**Data curation:** Wei Zhang, Jinxing Ji, Hongliang Zhang.

**Formal analysis:** Weipeng Liu, Hongliang Zhang.

**Investigation:** Lirong Deng, Hongliang Zhang.

**Methodology:** Yanfang Deng, Hongliang Zhang.

**Supervision:** Hushuang Yang, Hongliang Zhang.

**Validation:** Xingying Zheng, Hongliang Zhang.

**Visualization:** Siying Le, Hongliang Zhang.

**Writing – original draft:** Wei Zhang, Hongliang Zhang.

**Writing – review & editing:** Wei Zhang, Hongliang Zhang.
